# Traffic-Related Air Pollution and DNA Damage: A Longitudinal Study in Taiwanese Traffic Conductors

**DOI:** 10.1371/journal.pone.0037412

**Published:** 2012-05-21

**Authors:** Han-Bin Huang, Ching-Huang Lai, Guan-Wen Chen, Yong-Yang Lin, Jouni J. K. Jaakkola, Saou-Hsing Liou, Shu-Li Wang

**Affiliations:** 1 Graduate Institute of Life Sciences, National Defense Medical Center, Taipei, Taiwan; 2 Division of Environmental Health and Occupational Medicine, National Health Research Institutes, Zhunan, Miaoli, Taiwan; 3 Department of Public Health, National Defense Medical Center, Taipei, Taiwan; 4 Center for Environmental and Respiratory Health Research, Institute of Health Sciences, University of Oulu, Oulu, Finland; 5 Institute of Environmental Medicine, College of Public Health, China Medical University and Hospital, Taichung, Taiwan; Texas Tech University, United States of America

## Abstract

**Background:**

There is accumulating epidemiologic evidence that exposure to traffic-related air pollutants, including particulate matter (PM) and polyaromatic hydro carbons (PAHs), plays a role in etiology and prognosis of a large scale of illnesses, although the role of specific causal agents and underlying mechanisms for different health outcomes remains unknown.

**Objective:**

Our general objective was to assess the relations between personal exposure to traffic exhausts, in particular ambient PM_2.5_ and PAHs, and the occurrence of DNA strand breaks by applying personal monitoring of PM and biomarkers of exposure (urinary 1-hydroxypyrene-glucuronide, 1-OHPG) and effect (urinary 8-hydroxydeoxyguanosine, 8-OHdG and DNA strand breaks).

**Methods:**

We recruited 91 traffic conductors and 53 indoor office workers between May 2009 and June 2011 in Taipei City, Taiwan. We used PM_2.5_ personal samplers to collect breathing-zone particulate PAHs samples. Spot urine and blood samples after work shift of 2 consecutive days were analyzed for 1-OHPG, 8-OHdG and DNA strand breaks, respectively. Statistical methods included linear regression and mixed models.

**Results:**

Urinary 8-OHdG levels and the occurrence of DNA strand breaks in traffic conductors significantly exceeded those in indoor office workers in mixed models. Particulate PAHs levels showed a positive association with urinary 1-OHPG in the regression model (β = 0.056, p = 0.01). Urinary 1-OHPG levels were significantly associated with urinary 8-OHdG levels in the mixed model (β = 0.101, p = 0.023). Our results provide evidence that exposure to fine particulates causes DNA damage. Further, particulate PAHs could be biologically active constituents of PM_2.5_ with reference to the induction of oxidative DNA damages.

## Introduction

Traffic emissions include large quantities of carbon dioxide (CO_2_), carbon monoxide (CO), hydrocarbons (HC), nitrogen oxides (NOx), particulate matter (PM), and mobile source air toxics (MSATs), such as benzene, formaldehyde, acetaldehyde, 1,3-butadiene, and lead (where leaded gasoline is still in use). In addition, secondary by-products, such as ozone and secondary aerosols, such as nitrates and inorganic and organic acids, contribute to traffic-related air pollution [1]. There is accumulating epidemiologic evidence that exposure to traffic-related air pollutants plays a role in etiology and prognosis of a large scale of illnesses, including asthma, impaired lung function, allergy, adverse birth outcomes, cardiovascular disease and cancer, although the role of specific causal agents and underlying mechanisms for different health outcomes remains unknown [1].

Traffic is an increasingly important source of PM, especially fine PM with an aerodynamic diameter 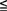
2.5 µm (PM_2.5_). Fine particles (PM_2.5_) can penetrate easily into respiratory tract and reach circulatory system, and be more toxic than coarse particles [2]. There is extensive epidemiologic evidence of the associations between both short- and long-term exposure to PM_2.5_ and the risk of respiratory and cardiovascular diseases, and adverse pregnancy outcomes.

Traffic is also the most important source of polycyclic aromatic hydrocarbons (PAHs) in urban ambient air [3], [4]. PAHs are ubiquitous constituents of urban airborne particles and are of major health concern mainly due to their well-known carcinogenic and mutagenic properties.

Biomarkers of exposure and outcome could be useful both in the quantification of the relations of interests as well as in providing insight into the specific causal agents and underlying biological mechanism.

Metabolites of pyrene in human urine can be measured as 1-hydroxypyrene (1-OHP) after deconjugation of the glucuronide with beta-glucuronidase or directly as 1- hydroxypyrene-glucuronide (1-OHPG) without deconjugation. Since 1-OHPG is approximately 5-fold more fluorescent than 1-OHP, it may provide a more sensitive biomarker for assessing exposure to pyrene in mixtures of PAHs [5], [6]. Furthermore, the relation between total PAHs and pyrene was highly correlated in the urban environment [7]. Thus, Urinary 1-OHPG has been used as a biomarker of exposure to PAHs [6], [8], [9].

Urinary 8-OHdG levels and DNA strand breaks could serve as biomarkers of early effects of exposure to PM, because there is evidence that exposure to ambient particulate matter (PM) induces oxidative stress, genotoxicity, and carcinogenicity [10]. Previous studies suggest that PM interacts with biological systems through direct generation of reactive oxygen species (ROS) from the surface of particles, organic chemicals, transition metals or other agitated processes in bodies and further contributes to the oxidative stress process [10], [11]. Besides, oxidative stress is defined as an imbalance between free radical production and antioxidant capacity resulting in excess oxidative products [12]. The spectrum of DNA-related oxidative products includes strand breaks, AP (apurinic/apyrimidinic) sites, and oxidized bases. With the latter group, attention has been focused on 8-OHdG, a major product with a clear mutagenic potential, which has been commonly used as a biomarker of oxidative stress in studies on ambient air pollution [9], [13]–[16]. On the other hand, DNA strand breaks can be measured by the comet assay and represent an early and critical step in chemical carcinogenesis [17].

Most previous epidemiological studies have based exposure assessment on data collected at stationary monitoring stations, which may introduce both random and systematic error in exposure assessment of individuals [18]. Furthermore, only a few studies that have collected personal PM mass or its components as measures of exposure have explored the related DNA damage effects [14]–[16].

Our general objective was to assess the relations between personal exposure to traffic exhausts, in particular ambient PM_2.5_ and PAHs, and the occurrence of DNA strand breaks. We assessed the personal exposure to ambient air PM_2.5_ concentration and the amount of PAHs bound to PM_2.5_, urinary 1-OHPG levels as a biomarker of exposure to PAHs, and urinary 8-OHdG concentration and the occurrence of DNA strand breaks as biomarker of effect. In order to best elaborate the relations of interest in existing environmental conditions, we selected our study population from the Taipei City traffic conductors who represent probably the most highly exposed population in Taiwan.

## Methods

### Study Population

We recruited 91 traffic conductors as the exposed group and 53 indoor office workers as the reference group aged 20 to 63 years between April 2009 and June 2011 in Taipei City, Taiwan. All subjects were free of cancer and pulmonary disease and all of them had been working in their current job position for at least 3 months.

### Ethics Statement

This study was approved by the Institutional Review Board of the National Health Research Institutes in Taiwan, and written informed consent from participants was obtained prior to study enrollment.

### Data Collection

The participants underwent health examinations and completed a self-administered questionnaire about demographic information, lifestyle habits, as well as history of previous and current diseases. Personal monitors were used to assess the PM_2.5_ concentration in the breathing zone for all particulates during the daily work shift. Post-shift urine and blood samples were collected from participants on 2 consecutive days.

### Air Sampling

The subjects were monitored for 9–10 hours per day equal to their working hours. Personal breathing-zone air samples were collected from the study subjects by battery-operated personal air-sampling pumps. The personal exposure monitors with a 2.5 µm impactor (The Personal Environmental Monitor, PEM; SKC Inc., Catalog No. 761–230, PA, USA) cut size was used in line with a Gilian GilAir 5 pump (Sensidyne Inc., Clearwater, FL) calibrated at a flow rate of 2 L/min. Particle samples were collected on Teflon filter (Biotech Line, Lynge, Denmark). Before and after sampling, the filters were weighed on a Micro Balance MT5 from Mettler-Toledo (Glostrup, Denmark) after conditioning for 24 h in a temperature- and relative humidity-controlled room. The mass collected in the filter was divided by the air volume sampled to calculate the gravimetric PM_2.5_ concentration.

### Quantification of PAHs in PM

The PAH analyses of air samples were conducted using methods of Tsai et al. [19]. Total particulate PAHs levels were determined as the sum of following 22 individual species: naphthalene, acenaphthylene, acenaphthene, fluorene, phenanthrene, anthracene, fluoranthene, pyrene, cyclopenta[c,d]pyrene, benz[a]anthracene, chrysene, benzo[b]fluoranthene, benzo[k]fluoranthene, benzo[e]pyrene, benzo[a]pyrene, perylene, indeno[1,2,3-cd] pyrene, dibenz[a,h]anthracene, benzo[b]chrysene, benzo[ghi]perylene, coronene, and dibenzo[a,e]pyrene. Recovery efficiencies determined by spiking known amounts of PAHs through the GC-MS analytic process ranged from 80% to 110%. The limit of detection of the 22 PAH compound fell to the range 19–386 pg/m^3^. Analyses of field blanks found no significant contamination (i.e., GC/MS integrated area < detection limit).

### Analysis of Urine Samples for 1-OHPG and 8-OHdG

The urine samples were collected within half an hour after the end of a work-shift. We divided the urine samples into several small volume aliquot and stored at −80°C freezer until analysis.

Urinary 1-OHPG was measured using the assay developed by Strickland et al. [5]. The recovery of the assay was 91%. The coefficient of variation of the assay was less than 5% during the period of sample analysis. The limit of detection was 0.05 ng/ml. Urinary 8-OHdG concentrations were measured using a liquid chromatography/MS/MS as described elsewhere [20]. A detection limit of 5.7 ng/L was obtained using seven repeated analyses of deionized water. The coefficients of variation in interday and intraday tests were <5%. Mean recovery of 8-OHdG in urine was 99%–102%. Each subject’s urinary 1-OHPG and 8-OHdG concentrations were normalized to urine creatinine.

### Single-cell Gel Electrophoresis (Comet assay)

The blood samples were collected in EDTA tubes within half an hour after the end of work-shifts. Blood (2 ml) from each subject was immediately stabilized with 2 ml of a 20∶80 (v/v) mixture of dimethyl sulfoxide (DMSO) and RPMI 1640 cell culture medium [21]. Aliquots of these samples were progressively frozen to −80°C for later analysis.

Comet assay procedure was performed according to manufacturer’s protocol (Trevigen, Gaithersburg, MD). Comet assay kits, reagents and slides were purchased from Trevigen (Gaithersburg, MD). In brief, we mixed 50 µl thawed blood with 500 µl molten LMAgarose (at 37°C), and 50 µl mixture was immediately pipetted onto CometSlide™. Slides were left at 4°C in the dark in refrigerator for 10 min. The slides were immersed in prechilled lysis solution. After 1 h in the dark at 4°C, the slides were immersed in freshly prepared Alkaline Solution (300 mM NaOH, 1mM EDTA, pH>13) for 1 hour and then electrophoresed (300 mA, 30 min). The process of the electrophoresis was performed in a cold room to diminish background damage. Once electrophoresis was completed, the slides were immersed twice in deionized water for 10 min each, then in 70% ethanol for 5 min. Samples were stored at room temperature in the dark with desiccant prior to scoring. We quantified the DNA damage of 100 randomly selected leukocytes (50 cells from each duplicate well) after staining with 20 µl SYBR Green, using Pixera Penguin 150CL Cooled CCD digital camera systems (Pixera, USA) attached to a fluorescent microscope (Olympus, Japan). The percentage of DNA in tail (%T) was used as a measure of DNA damage and computed using the Komet Software version 5.5 (Kinetic Imaging Ltd, Liverpool, UK).

Negative and positive control cells purchased from Trevigen (Trevigen, Gaithersburg, MD) were included in the assay as a quality control. The percentage of DNA in tail for the negative and positive controls were <10% and >30%, respectively. Samples were coded and run along with quality control samples in the same bench.

### Statistical Methods

The levels of PM_2.5_, particulate PAHs, urinary 1-OHPG, urinary 8-OHdG and DNA strand breaks were compared between the exposed and reference groups by Mann-Whitney U test. The distribution of urinary 1-OHPG, urinary 8-OHdG and DNA strand breaks by order of day within the exposed and reference groups were compared using Wilcoxon signed rank test. Multiple linear regression models were used to assess the relations between PM_2.5_ levels, particulate PAHs and urinary 1-OHPG adjusting for the covariates (such as age, gender, education level, smoking habit, season of data collection and exposure status).

Mixed-model repeated measures analysis (Proc mixed) was used to investigate the relations between urinary 1-OHPG, 8-OHdG and DNA strand breaks after adjusting for fixed covariates. These models treated the subjects as random effect and model selections were based on Akaike’s Information Criterion. The compound symmetry and variance components were constructed as the covariance structures. The dependent variables were transformed by the natural logarithm. Residual and influence analyses were conducted. To calculate the predictive value of an X unit increase in one of the predictors, the following formula was used: [exp^(model estimate* X)^ − 1] * 100. A two-sided p-value <0.05 was considered statistically significant. All statistical analyses were performed using SAS (version 9.1.3; SAS Institute Inc., Carry, NC, USA.).

## Results

### Study Population

Characteristics of the study population were compared between the exposed and reference groups (table 1). The mean age was 49.2 years (SD 9.17) in the exposed group and 42.9 years (SD 8.86) in the reference group. The exposed group had a lower percentage of women and a lower educational level than the reference group. The most common season for data collection was spring for the exposed group, but spring and winter for the reference group. The distributions of lifestyle factors, such as smoking habit, drinking alcohol and cooking habit, were similar between the exposed and reference groups.

**Table 1:** Characteristic of subjects in exposed and reference groups.

	Exposed group		Reference group		
Variables	(N = 91)		(N = 53)		p-value^a^
	N	%	N	%	
Age (years) (Mean±SD)^b^	49.19±	9.17	42.85±	8.86	<0.001
Gender					
Male	68	74.7	12	22.6	
Female	23	25.3	41	77.4	
BMI (Kg/m^2^) (Mean±SD) b	25.11±	3.55	29.06±	6.60	<0.001
Educational level					<0.001
High school	57	64.0	9	17.0	
College	32	36.0	44	83.0	
Current smoker					0.128
No	70	76.9	47	88.7	
Yes	21	23.1	6	11.3	
Drinking alcohol					1.000
No	74	83.1	43	82.7	
Yes	15	16.9	9	17.3	
Vitamin supplement					0.140
No	39	43.3	30	57.7	
Yes	51	56.7	22	42.3	
Cooking habit					0.122
No	51	59.3	22	44.0	
Yes	35	40.7	28	56.0	
Season of data collection^c^					0.027
Spring	48	52.7	20	37.7	
Summer	14	15.4	3	5.7	
Fall	11	12.1	12	22.6	
Winter	18	19.8	18	34.0	

a χ^2^ test.

b Student’s t test.

c Fisher’s exact test.

### Comparison of PM_2.5_ Level, Particulate PAHs, Urinary 1-OHPG, Urinary 8-OHdG and DNA Strand Breaks between the Exposed and Reference Groups

Table 2 shows that the distributions of PM_2.5_ exposure and particulate PAHs in the exposed and reference groups. The median levels of PM_2.5_ and particulate PAHs in the exposed group were higher than those in the reference group. Spearman correlation between PM_2.5_ and particulate PAHs levels was significantly positive in the traffic conductors (r = 0.42, p<0.01), but not in the indoor office workers (r = −0.08, p = 0.57) (data not shown).

**Table 2:** Concentrations of PM_2.5_ and particulate polycyclic aromatic hydrocarbons (PAHs) for the exposed and reference groups.

	Exposed group		Reference group		
Variables	(N = 91)		(N = 53)		p-value^a^
	Median(Q_25_-Q_75_)	N	Median(Q_25_-Q_75_)	N	
**External exposure**					
PM_2.5_ (µg/m^3^)	82.87 (63.11–134.15)	91	70.82 (56.31–99.25)	50	0.054
Particulate PAHs(ng/m^3^)	13.07(8.76–20.55)	91	8.24 (6.87–8.76)	50	<0.001

a Mann-Whitney U test.

Table 3 shows the concentrations of urinary 1-OHPG, urinary 8-OHdG and DNA strand breaks in the exposed and reference groups by order of day. There were no significant differences of urinary 1-OHPG, urinary 8-OHdG, and DNA strand breaks between 1^st^ day and 2^nd^ day in the exposed and reference groups. In terms of urinary 1-OHPG, 8-OHdG and DNA strand breaks, the median level in the exposed group was significantly higher than those in the reference group both in the first and second day.

**Table 3:** Concentrations of urinary 1-OHPG, 8-OHdG and DNA strand breaks in the exposed and reference groups by order of day.

	Exposed group					Reference group						
Variables	1^st^ day		2^nd^ day		p-value^a^	1^st^ day		2^nd^ day		p-value^b^	p-value^c^	p-value^d^
	Median(Q_25_-Q_75_)	N	Median(Q_25_-Q_75_)	N		Median(Q_25_-Q_75_)	N	Median(Q_25_-Q_75_)	N			
**Biomarker of exposure**												
Urinary 1-OHPG (µg/g creatinine)	0.36 (0.21–0.58)	83	0.33 (0.17–0.55)	81	0.393	0.16 (0.08–0.23)	47	0.18 (0.11–0.31)	46	0.634	<0.001	0.001
**Biomarkers of effect**												
Urinary 8-OHdG (µg/g creatinine)	3.67 (2.72–5.01)	87	3.44 (2.66–4.30)	83	0.494	2.53 (1.58–3.59)	47	2.28 (1.53–3.50)	46	0.133	<0.001	<0.001
DNA strand breaks (%)	14.95 (12.65–18.53)	89	15.37 (12.85–19.63)	82	0.314	9.26 (7.64–10.62)	49	9.46 (8.02–11.20)	47	0.536	<0.001	<0.001

a Wilcoxon Signed rank test within exposed group.

b Wilcoxon Signed rank test within reference group.

c Mann-Whitney U test between exposed and reference group for the first day.

d Mann-Whitney U test between exposed and reference group for the second day.

### Relations between Environmental Exposure and Biomarker of Exposure

**Figure 1:**
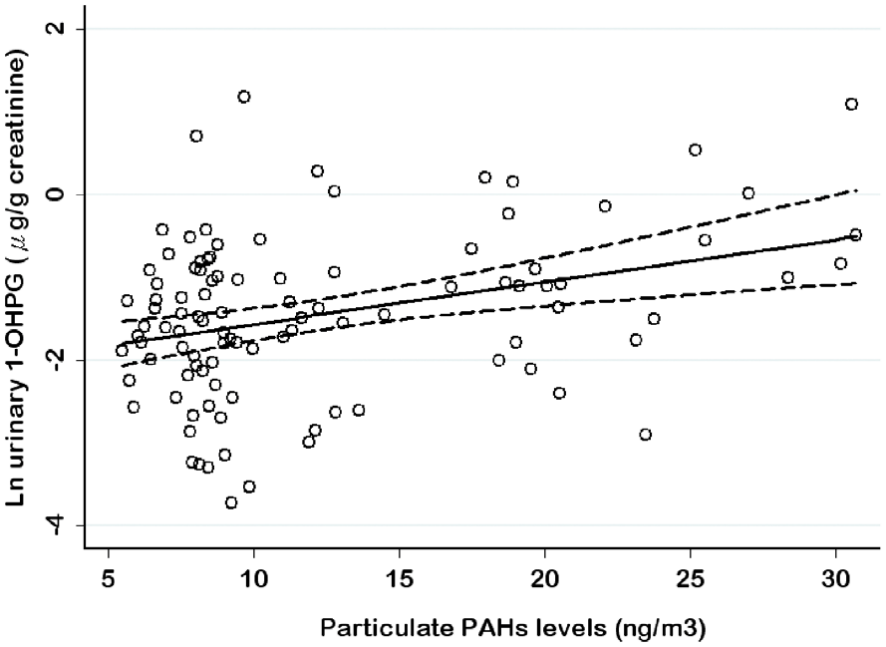
The scatter plot of urinary 1-OHPG levels vs. particulate PAHs levels in nonsmoking workers.

Table 4 presents associations between PM_2.5_ levels, particulate PAHs and urinary 1-OHPG levels in all workers and nonsmoking workers. In the adjusted model, there was no significant association between PM_2.5_ levels and urinary 1-OHPG levels in all workers and nonsmoking workers. In contrast, we found a significant positive association between particulate PAHs and urinary 1-OHPG in both groups of workers after adjusting for fixed covariates (p<0.05). That is, for a 10 ng/m^3^ increment of particulate PAHs level, a 75% increment in urinary 1-OHPG was observed among nonsmoking workers. Figure 1 illustrated a scatter plot of urinary 1-OHPG levels by particulate PAHs levels using regression models in nonsmoking workers after adjusting for other covariates with 95% confidence interval.

### Relations between Biomarkers of Exposure and Effect

**Figure 2:**
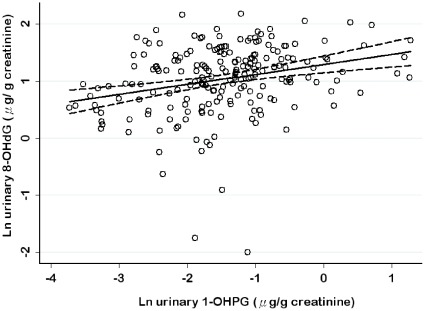
The scatter plot of urinary 8-OHdG levels vs. urinary 1-OHPG levels in nonsmoking workers.

Table 5 presents the results of linear mixed models for determinants of urinary 1-OHPG, urinary 8-OHdG and DNA strand breaks in nonsmoking workers. After adjustments were made for other covariates, we found that an increase in urinary 1-OHPG was significantly related to an increase in urinary 8-OHdG (p<0.05). That is, for a 75% increment of urinary 1-OHPG levels, a 7.6% increment in urinary 8-OHdG levels was observed. The exposure at group level was a significant determinant of urinary 1-OHPG levels, urinary 8-OHdG levels and DNA strand breaks. However, urinary 1-OHPG and 8-OHdG levels were not significant determinants of DNA strand breaks. On the other hand, age was a significant determinant of urinary 8-OHdG levels and DNA strand breaks. Season was a significant determinant of urinary 8-OHdG levels. Females had on average higher levels of urinary 1-OHPG than males in the adjusted models. Figure 2 illustrated a scatter plot of urinary 8-OHdG levels by urinary 1-OHPG levels using mixed models in nonsmoking workers after adjustment for other covariates and presents graphically the lower and upper limits of the 95% confidence interval.

**Table 4:** Relations of PM_2.5_ exposure and particulate PAHs with urinary 1-OHPG by regression models.

	Ln urinary 1-OHPG(µg/g creatinine)		
Variables	β	95% CI	p-value
**Total workers (N = 144)**			
Environmental exposure^a^			
PM_2.5_ (µg/m^3^)	−0.0003	−0.003 to 0.002	0.819
Environmental exposure^a^			
PM_2.5_ (µg/m^3^)	−0.0006	−0.004 to 0.002	0.675
Particulate PAHs (ng/m^3^)	**0.043**	**0.011 to 0.075**	**0.010**
**Nonsmoking workers (N = 117)**			
Environmental exposure^b^			
PM_2.5_ (µg/m^3^)	−0.0007	−0.004 to 0.002	0.626
Environmental exposure^b^			
PM_2.5_ (µg/m^3^)	−0.001	−0.004 to 0.002	0.503
Particulate PAHs (ng/m^3^)	**0.056**	**0.014 to 0.098**	**0.010**

a Adjusted for age, gender, educational level, smoking habit, season of data collection, and group.

b Adjusted for age, gender, educational level, season of data collection, and group.

## Discussion

Our results provide evidence that exposure to particulate PAHs in PM_2.5_ is a determinant of urinary 1-OHPG levels; urinary 1-OHPG could thus serve as a biomarker of ambient exposure to PAHs. Further, urinary 8-OHdG levels are related to urinary 1-OHPG levels and thus urinary 8-OHdG has potential to serve as a biomarker of effect. An increase of 10 ng/m^3^ in particulate PAHs was associated with a 75% increase in urinary 1-OHPG levels and a 75% increase in urinary 1-OHPG levels was associated with a 7.6% increase in urinary 8-OHdG levels. The results provide evidence that exposure to ambient air PM increases the occurrence of DNA damage, and exposure to particulate PAHs increases urinary 8-OHdG levels.

**Table 5:** Determinants of urinary 1-OHPG, 8-OHdG and DNA strand breaks in nonsmoking workers by mixed models (No^a^ = 234).

	Ln urinary 1-OHPG (µg/g creatinine)		Ln urinary 8-OHdG (µg/g creatinine)		Ln DNA strand breaks (%)	
Variables	β (95% CI)	p-value	β (95% CI)	p-value	β (95% CI)	p-value
Age (year)	0.009 (−0.009 to 0.027)	0.328	0.015 (0.003 to0.026)	**0.016**	−**0.008 (**−**0.014** **to** −**0.001)**	**0.019**
Gender						
Female (reference)						
Male	−**0.369 (**−**0.712** **to** −**0.026)**	**0.035**	−0.111 (−0.337 to 0.116)	0.334	0.035 (−0.083 to 0.153)	0.557
Educational level						
High school (reference)						
College	−0.056 (−0.322 to 0.435)	0.770	0.124 (−0.370 to 0.124)	0.323	0.122 (−0.253 to 0.010)	0.069
Group						
Control group (reference)						
Exposed group	**0.634 (0.228 to 1.040)**	**0.003**	**0.379 (0.108 to 0.649)**	**0.007**	**0.621 (0.477 to 0.765)**	**<0.001**
Season						
Winter (reference)						
Spring	0.125 (−0.222 to 0.472)	0.477	−0.256 (−0.483 to −0.029)	0.027	0.023 (−0.097 to 0.143)	0.701
Summer	0.080 (−0.457 to 0.616)	0.769	−0.506 (−0.857 to −0.156)	0.005	0.051 (−0.143 to 0.245)	0.603
Fall	0.083 (−0.385 to 0.550)	0.727	−0.237 (−0.542 to 0.067)	0.125	0.094 (−0.065 to 0.253)	0.242
Ln 1-OHPG (µg/g creatinine)			**0.101 (0.014 to 0.187)**	**0.023**	0.007 (−0.036 to 0.051)	0.732
Ln 8-OHdG (µg/g creatinine)					−0.019 (−0.088to 0.050)	0.587

a Abbreviations: number of observations (No).

We hypothesized that particulate PAHs in PM could be a stronger determinant of urinary 8-OHdG compared with PM mass alone. Recent literature suggests that some chemical components of PM may have stronger effects than others and the differences in effect estimates across cities or seasons may be related to the chemical composition of particles [22], [23]. Particulate PAHs can be converted to PAH-quinones by cytochrome P450, epoxide hydrolase and dihydrodiol dehydrogenase [24], [25]. It has been reported that compounds having a quinone-structure are able to produce ROS in redox cycle [26], [27]. Laboratory-based *in vitro* or *in vivo* studies have consistently reported significant associations between 8-OHdG and PAHs. However, an early study found a dose-response relationship between PM_2.5_ -bound metal and urinary 8-OHdG among boiler markers at boiler making plants [14]. Wei et al. [16] reported that PM_2.5_ mass, PAHs and metal were significantly associated with an increased levels of urinary 8-OHdG after work-shift. Another panel study reported that the personal PM_2.5_ exposure was a predictor of 8-OHdG in lymphocytes with an 11% increase in 8-OHdG per 10 µg/m^3^ increase in PM_2.5_ concentration [15]. These studies showed that PM_2.5_ or its components could increase the burden of oxidative stress.

We found no associations between neither urinary 1-OHPG levels nor urinary 8-OHdG levels and DNA strand breaks, which was contrary to our hypothesis but in agreement with a previous study [28]. A recent study also reported that different PAH industries, such as graphite electrode, refractory, and coke oven, could contribute to genotoxic DNA damage and DNA damage was not unequivocally associated to PAH on the individual level most likely due to potential contributions of co-exposures [29]. Moreover, our finding of the exposed group status as an independent predictor of DNA strand breaks is consistent with an idea that traffic conductors could also be exposed to other unmeasured hazards, such as benzene and ozone. Taken together, these observations suggest that while particulate PAHs may have the potential to induce DNA strand breaks, other factors probably play a role in the response of the organism to environmental pollution.

Urinary 8-OHdG levels as not a significant predictor of DNA strand breaks indicated that 8-OHdG levels could play a minor role in the process of DNA strand breaks, and other spectrum of DNA damage could mainly contribute to increase the levels of DNA strand breaks. Actually, DNA damages based on alkaline single-cell gel electrophoresis include not only DNA strand breaks but also base modifications, as the oxidized purine bases (8-OHdG and others) and pyrimidine bases could be converted into additional DNA single strand breaks [17], [30]. Therefore, the origin of direct strand breaks and alkali-label sites that may include modified sugar and base residual is difficult to identify using alkaline comet assay. Apparently, this strongly depends on the DNA modifying agent.

There was a significant association between the ambient concentrations of PM_2.5_ and particulate PAHs exposure in the traffic conductors while such association was not present in the office workers. These differences between the occupational groups may be related to the outdoor and indoor locations that reflect different working conditions, as well as to differences in the dispersion of toxic substances between the outdoor and indoor workplace. PM_2.5_ levels and particulate PAHs levels of traffic conductors were lower than those in Taipei toll station workers (range from 87–346 µg/m^3^ for PM_2.5_ and 480–6026 ng/m^3^ for particulate PAHs) [7], [31]. This may be due to lower traffic intensities and different pattern of outdoor working activities between toll-station workers and traffic conductors.

As to the effect of age on urinary 8-OHdG, our study result was consistent with a previous finding of a significantly increasing trend in urinary 8-OHdG concentrations by increasing age [32]. Previously younger workers were reported to have higher levels of DNA strand breaks than older workers due to higher physical-activity loading on job [33], [34]. In the present study, the highest concentrations of urinary 8-OHdG were measured during the winter, which corresponds to higher levels of PAHs exposure in the winter than during other seasons [4]. Gender-related differences in urinary 1-OHPG levels could be explained by gender differences in endogenous metabolism of PAHs [35], [36].

Our study has some limitations. First, both traffic-related sources and other indoor sources contribute to the total exposure to PM and PAHs, and therefore, our exposure assessment was likely to underestimate personal PM_2.5_ and particulate PAHs exposures from traffic-related sources. Secondly, we did not assess exposure to other air pollutants, such as carbon monoxide, nitrogen dioxide and ozone. If these pollutants are associated with particulate matter, the estimated effects could be confounded. Last, genetic differences in PAH-metabolizing enzymes and DNA repair in the study population could affect the estimated effects. However, repeated measurement design in our study could decrease the influence of genetic background within-subject.

Our results provide new evidence that exposure to fine particulates may cause DNA damage by showing associations between measured ambient PAHs bound to PM_2.5_ and urinary 1-OHPG biomarkers as a biomarker of exposure and between urinary 1-OHPG and urinary 8-OHdG as a biomarker of DNA damage. In addition, our comparison of traffic conductors and office workers indicates that exposure to traffic exhausts could increase the amount of DNA strand breaks. These findings are consistent with the hypothesis that exposure to traffic exhausts increases carcinogenic and mutagenic processes in humans, which support results from previous epidemiologic studies [18], [37].

In summary, this is the first study to indicate that the elevated levels of particulate PAHs bound to PM_2.5_ could increase urinary 8-OHdG levels through the elevated levels of urinary 1-OHPG. These results provide an indication that air pollution PM could be associated with DNA damage, and particulate PAHs could be the biologically active constituent of PM with regarding to the induction of oxidative DNA damage.
